# The Regulation of Telomerase in Oncogenesis

**Published:** 2009-04

**Authors:** D. A. Skvortzov, M. P. Rubzova, M. E. Zvereva, F. L. Kiselev, O. A. Donzova

**Affiliations:** 1 Department of Chemistry, Moscow State University, 119992 Moscow;; 2 Blokhin Oncological Science Centre, Russian Academy of Medical Science, 115478 Moscow

## Abstract

The influence that the expression of the human (glial-derived neurotrophic factor (GDNF)) neurotrophic factor has on the morphology and proliferative activity of embryonic stem cells (SC) of a mouse with R1 lineage, as well as their ability to form embroid bodies (EB), has been studied. Before that, using a PCR (polymerase chain reaction) coupled with reverse transcription, it was shown that, in this very lineage of the embryonic SC, the expression of the receptors' genes is being fulfilled for the neurotropfic RET and GFRα1 glia factor. The mouse′s embryonic SC lineage has been obtained, transfected by the human GDNF gene, and has been fused with the "green" fluorescent protein (GFP) gene. The presence of the expression of the human GDNF gene in the cells was shown by northern hybridization and the synthesis of its albuminous product by immunocitochemical coloration with the use of specific antibodies. The reliable slowing-down of the embriod-body formation by the embryonic SC transfected by the GDNF gene has been shown. No significant influence of the expression of the GDNF gene on the morphology and the proliferative activity of the transfected embryonic SCs has been found when compared with the control ones.

In 1961 Hayflick and Moorhead showed that a somatic cell culture has a limited life span [1]. In 1973 Olovnikov suggested that shortening the chromosomal ends (telomeres) determines the potential number of cell divisions. [2]. Telomeres protect the cellular genome from degradation; they participate in the chromosomal pairing during meiosis and in the gene expression regulation in the telomeres region [3]. In immortal cells that can divide infinitely, this should be the mechanism for compensating the chromosomal shortening. In 1975 Blackburn and Greider discovered the enzyme telomerase that elongates chromosomes [4].

Telomerase is a ribonucleoprotein complex that consists of components that are absolutely required for its activity: the RNA molecule and Telomerase Reverse Transcriptase TERT [5]; also, optionally several telomerase-associated proteins could be included in the telomerase complex. TR is also a template for TERT when telomerase elongates telomeres. Telomerase exists in human cells as dimers and contains two subunits of reverse transcriptase and two RNA molecules [6]. In human telomerase, p23/p90-shaperone, which is responsible for the complex assembling/configuration, binds 14-3-3, which is responsible for nuclear localization, and TP1 with an unknown function. Proteins hGAR1, Dyskerin/NAP57, hNHP2, and C1/C2, which are responsible for the stability, maturation, and localisation of RNA, bind to the hTR; La and hStau, which are supposedly responsible for the binding to telomeres; L22, which acts in processing and nuclear localisation; and hNOP10, A1/UP1, and TP1 with an unknown function [7]; TCAB1, which is responsible for the localisation of hTR in Cajal bodies and binding with telomers [8]. The enzymatic activity of human telomerase in the rabbit reticulocytes lysate is detected by adding hTR and hTERT [9, 10]. Note that telomerase functioning in vivo is not always consistent with the telomerase activity that was measured in vitro. For example, adding the Hemagglutinin epitope to the C-end of hTERT stops telomere alongation but does not suppress telomerase activity [11].

Telomerase activity detected in vitro appears in leucocytes in the G1 phase (Fig. 1) [12]. On the other hand, telomeres are replicated in vivo during the S-phase (Fig. 1) [13, 14]. During most of the cell cycle, TCAB1 helps the hTR accumulation in Cajal bodies [8], and in S-phase it is combined with telomeres in a cell. During the S-phase of the cell cycle, hTERT also moves to telomeres [15, 16]. This means that there is regulation at the level of the spatial localisation of active telomerase (enzyme) and telomere (substrate). A correlation between the telomerase activity and the length of telomeres is not always possible to obtain. For example, there is no dependency between the length of telomeres and telomerase activity in leukaemia [17]. 

**Fig. 1.**
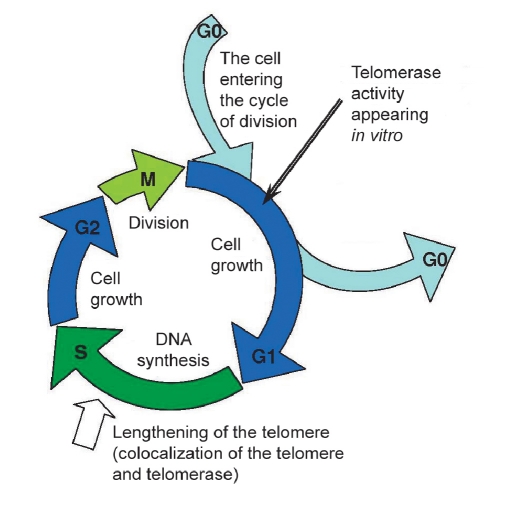
Cell cycle scheme. The appearance of telomerase activity in vitro happens in the G1-phase, but it works at the S-phase.

The two-step hypothesis of cell aging and immortalisation M1/M2 theory describes the activity of telomerase dependency on the number of cell divisions very well (Fig. 2). In embryonic cell lines, telomerase is active and the length of telomeres is constant. In stem cells, the activity of polymerase is lower and it only partially compensates the telomeres shortening. In somatic cells, telomerase in not active. The shortening of telomeres leads to the moment of M1, i.e., the achievement of the Hayflick limit (point A on Fig 1) and the transition of cells to the senescence (aging) condition, which could be rescued by the inactivation or deletion of pRB/p16 or p53. Cells that pass through M1 continue their cell division and achieve the condition of crisis M2 (point B on Fig 2), which leads to massive cellular death. Cells that survive begin their transformation into cancer cells. Cancer cells have the ability for unlimited cell division and support the length of telomeres (usually due to telomerase activity). In the case of the transfection of hTR-expressing somatic cells by the hTERT gene (point C on Fig. 2) before the moment of M2, they, similarly to cancer cells, demonstrate elongation and stabilization of telomeres [7].

**Fig. 2.**
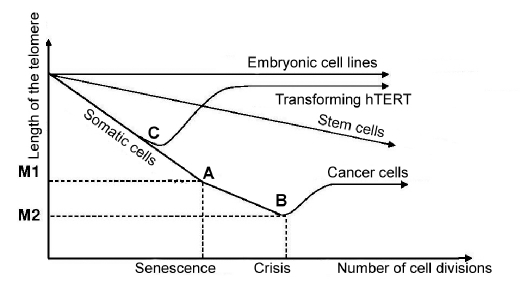
Dependence of telomeres length on the cell cycle number in different types of cells: embryonic cell lines, somatic cells, and hTERT-transfected cells. (A) achieving the Hayflick limit by cells, (B) crisis with the following cell death and transformation of surviving cells into cancer cells, (C) the transfection of cells by the hTERT gene.

Telomerase activity, with rare exceptions, does not occur in human somatic cells and tissues. Its activity was shown in reproductive tissues, as well as in intensively renewing tissues, such as some types of blood cells, the intestinal epithelium, and the layer of skin cells [18]; however, the level of telomerase activity in the somatic cells with active polymerase is lower than in cancer cells [19].

## The frequency of telomerase activity detection in different types of tumors

Telomerase is active in most (80-90%) tumor cells (Table 1), and this activity is the main instrument for supporting the telomeres length. There are non-malignant types of tumors and other types of non-cancer lesions that demonstrate telomerase activity almost 100% of the time, but there are others without any activity [19-21]. Tumor cell of some cell types can use an alternative mechanism of telomeres length support that is based on recombination [22]. In case of the transaction of cells with an alternative telomere-supporting mechanism by the gene hTERT, both mechanisms are active; however, in the case of the hybridization of cell lines with different mechanisms, telomerase is present in hybrids, and indications of an alternative mechanism of telomere lengthening disappear [23]. It should be noted that telomerase itself is not oncogene. Cell lines that were tranfected by the hTERT gene do not demonstrate indications of malignant transformation for a long time [24, 25].

**Table 1:** Frequency of telomerase activity in different types of tumors.

Type of tumor	Frequency of telomerase activity , %	Number of researched samples	[Reference]
MALIGNANT TUMORS:			
Small-cell lung cancer	100	15	[21]
	90	10	[30]
Non-small-cell lung cancer	78	125	[21]
	83	68	[31]
	84	32	[30]
phlegm from lung cancer patients	67,6	34	[30]
Stomach cancer	72	85	[21]
Colon cancer	89	138	[21]
Pancreas cancer	95	43	[21]
Liver cancer	86	173	[21]
	79	24	[32]
Mammary gland cancer	88	339	[21]
	59	44	[33]
Cervical cancer	100	16	[21]
Ovarian cancer	91	23	[21]
Prostate cancer	90	58	[21]
Kidney cancer	83	115	[21]
William cancer	100	6	[21]
	95,7	164	[43]
Retinoblastoma	50	34	[21]
Neuroepithelial tumors	62	107	[35]
Glioblastoma	75	60	[21]
	72	47	[35]
	28	25	[36]
	26	38	[37]
Astrocytoma, II stage	20	15	[35]
Oligodenryogliomas	100	19	[21]
	100	4	[35]
Oligodenryoglioma, II stage	14	14	[37]
Anaplastic oligodenrioglioma	43	7	[37]
Anaplastic astrocytoma	10	20	[21]
	40	15	[35]
	23	13	37
Neurobalstoma	94	100	[38]
Melanoma	86	7	[21]
Squamous cell carcinoma	83	18	[21]
Basalioma	95	77	[21]
HEMOBLASTOSES:			
Lymphima, low level of malignancy	86	14	[21]
Lymphima, high level of malignancy	100	16	[21]
Malignant lymphoma of CNS	83	12	[39]
Myelodisplastic syndrome	67	6	[21]
Cgronic myeloleucosis	71	42	[21]
Cgronic myeloleucosism, acceleration phase	100	21	[21]
Cgronic myeloleucosis, early stage	14	14	[21]
Cgronic myeloleucosis, late stage	57	7	[21]
Acute myeloblastic leucosis	73	64	[21]
Acute lymphoblastic leucosis	80	5	[21]
Lymphogranulematosis (lymphoid predominance)	63,6	33	[40]
Lymphogranulematosis (nodular sclerosis )	89,7	39	[40]
Lymphogranulematosis (mixed-cells variant)	96,1	26	[40]
Lymphogranulematosis (lymphoid exhaustion)	100	7	[40]
NON-MALIGNANT DISEASES:			
Adenoma of colon	45	44	[21]
Hepatitis/liver cirrhosis (activity is weaker than in case of liver cancer)	29	148	[21]
	8/24	24/34	[32]
	25/45,9	80/37	[41]
Mammary gland fibroadenoma	75	12	[21]
Leiomyoma	0	14	[21]
Meningomyoma	0	25	[36]
Non-malignant lesion of lymphatic vessel	33	15	[21]
Non-malignant lesion of amygdalae	100	23	[21]

Telomerase activity could appear as a result of clone selection in the situation of critical shortening of telomeres [26] (Fig. 2, position B). Firstly, cells start to divide intensively, and the telomeres shorten; however, only cells with telomerase activation survive. In this case telomerase activity could be a marker of malignant progression and negative prognosis. For instance, the main increase of telomerase activity during lymphogranulomatosis appears at the transition between the first and second stages [27]. In other possible scenarios, telomerase activity appears at the same time as other cancer-leading metabolic abnormalities as a result of original cell damage. In this case telomerase activity appears just at the beginning of a disease, and it could be a good marker of the beginning of oncological processes. For example, there is no dependency between telomerase activity and the stage of cancer during cervical carcinoma; telomerase is active even at the first stage, and its activation occurs in pre-tumor illnesses [28]. Telomerase could be active originally in the cell types of interest, and this activity just becomes stronger during the transition to cancer; e.g., as happens during hemoblastosis [26]. Telomerase also could be originally active in the case of the transformation of stem cells [29]. In this case telomerase activity will be obtained at the beginning of tumor growth, because the method of its detection does not allow the activity from the single cell from the surrounding tissue to be seen, but even a small pool of telomerase-positive cells will be detected. 

Unfortunately, most publications provide information only about the presence of telomerase activity in certain types of cancer. The mechanisms of telomerase activation are usually investigated in a cell culture, and it is not often possible to conclude by which mechanism and how often they are met in the cancer under study in vivo. 

## The Amplifications of hTR and hTERT Genes

The hTR gene has one copy and is located in the Chromosome 3 at the position 3q26.3. This chromosomal region is amplified, for example, in the case of cervical cancer, lung cancer, and squamous cell carcinoma of the head and neck. The number of copies of the hTR gene increases in tumor cells more than in normal cells, and, correspondingly, the hTR expression increases in the case of cervical cancer, lung cancer, and squamous cell carcinoma of the head and neck [42].

The hTERT gene is located in Chromosome 5 at the position 5q15.33 in the region that is also amplified in some types of cancer [42]. Because the amplification of telomerase genes occurred during the amplification of chromosomes with those genes but not in the locus-specific amplification, it is possible to conclude that this process is nonspecific. In the case of cervical squamous cell carcinoma, the expression of hTERT is not related to the amplification of hTERT [43]. On the other hand, this amplification could possibly occur as a result of the chromosomal instability and aneuploidy that happens during the critical shortening of telomeres. 

## Regulation of TERT Transcription

hTERT transcription is very low or not detected at all in most human tissues, but it often appears in these tissues after neoplastic transformation [44].

## Structure of the telomerase reverse transcriptase promoter 

The hTERT promoter does not contain TATA or CAAT, which are typical for the binding of RNA-polymerase II, and it is GC-rich. There are different data on the position of the region of the transcription′s initiation. Note that, now and further in this paper, we use the numeration of nucleotides (point + 1 bp (nucleotide base pairs)) from point A in the triplet ATG, which is the beginning of translation. It was shown [45] (using the method of anti-RNAse protection) that there are several protected regions in different hTERT-positive cell lines, which indicates the possibility of several sites of transcription initiation (in the region of from -40 to 100 bp). The most commonly protected region in the region of transcription initiation is at the position -55 G from the beginning of translation. It was shown [46] by analyzing caped mRNA from the HeLa cell line that the site of transcription start is at position -77 bp. Recently, most authors have accepted this view. 

Sites that are responsible for the regulation of hTERT transcription are located in the region of 2000 bp before the sites of translation and transcription initiation [47, 48]. The most important site for activation is the region from 250-300 bp before ATG to tens of bp after it [46]. Apart from this, the GC-rich region of the promoter forms CpG-islands near the ATG, which indicates that methylation could take part in the regulation hTERT expression [7]. 

**Fig. 3.**
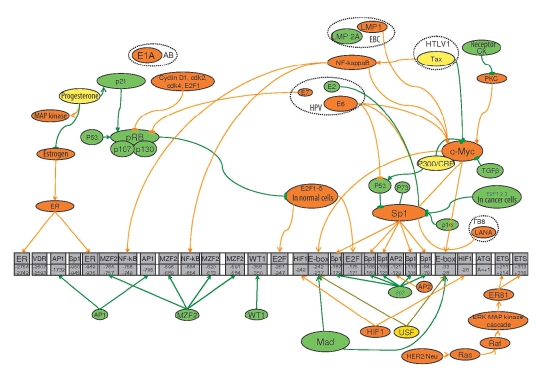
The scheme of the effect of hTERT- promoter's transregulators. The inhibitors of the hTERT promoter are green. Activators are orange. Double-action effectors are yellow. → points to the activation of the following player of cascade, †is the inhibition of the following cascade player, and the absence of an arrow means co-action.

The activity of the promoter is related to the distribution of its regions for the binding of regulatory proteins, which do not interfere with the promoter, but also the transmitters of the effects of other regulators. Figure 3 shows the scheme of the influence in different effectors on the hTERT promoter; this will be considered further in detail. 

### The methylation of the hTERT promoter

In tumor cells, the mutilation profile is different from the mutilation profile of normal cells. An analysis of the telomerase promoter has shown two CpG-islands; one of them is -900 bp from the start codon ATG [49].

In the case of cancer cell lines and intestinal cancer tissues, as has already been shown, there is a hTERT expression and CpG-islands of the hTERT promoter are fully or partially mutilated [50]. After extended treatment with the demethylation agent 5-aza-2'-deoxicitidine (5azadC) of cell lines Lan-1, HeLa, and Co115, in which telomerase is active and the hTERT promoter is hyperventilated, a 95% decrease in the level of hTERT-promoter methylation, and a decrease of the level of the hTERT mRNA expression were shown. Telomerase activity strongly decreases after 2-4 passages of cells in the presence of 5azadC [51].

The markers of active chromatin and telomerase expression are detected when the region of hTERT-promoter located from -73 to +227 bp around the transcription site is not methylated [52]. In normal cells, the hTERT-promoter hypermethylation suppresses telomerase activity and hTERT-mRNA expression, and, after treatment by 5azadC, telomerase starts to be activated [53]. Analysis of hTERT-promoter methylation in patients with chronic B-cells lymphoid leucosis has shown that they have high telomerase activity but a low level of methylation [49]. There is no direct correlation between telomerase activity and the hTERT-promoter methylation status for cell lines of ostecarcoma, ovarian cancer tissue samples, cervical cancer, and normal tissues [54, 55].

This data variation indicates that DNA methylation is not a critical factor for telomerase expression regulation in cancer. It occurs together with other disturbances of the system of hTERT-promoter regulation. Apart from this, both methylation and demethylation could affect telomerase transregulators, but not the promoter itself. 

### The Methylation of histones of the hTERT promoter

Histone methylation, which is provided by methyltransferases and demethylases, plays an important role in the regulation of the chromatin structure and transcription. Methyltransferase SMYD3, which is involved in oncogenesis, specifically activates hTERT. This methyltransferase binds to its specific binding region on the hTERT promoter and the thrice methylated H3-K4 histone. Suppressing SMYD3 in cancer cells stops H3-K4 threemethylation, makes the binding between promoter and trans-activators Sp1 and c-Myc weaker, and leads to the decreased acetylation of H3 histone in the hTERT-promoter, which leads to a decrease in hTERT-mRNA and a decrease of telomerase activity [56].

### The acetylation and deacetylation of hTERT-promoter's histones 

Acetyltransferases GCN5 and Tip60 acetylate histones H3 and H4, which leads to the activation of hTERT-trancription. The same acetyltransferases acetylate Myc, which leads to a decrease of its degradation [57] and other proteins that are involved in gene transcription, which could nondirectly regulate the hTERT promoter. Acetyltransferase p300 and the accompanying CBP are transcription coactivators which interact with a lot of sequence-specific transcription factors, and they are coactivators of onco-suppressor p53 [58]. p300 is a coactivator of hTERT transcription, and the c-Myc protein brings this protein to the promoter. Even p300 and CBP can stabilize Myc in an acetylation-independent manner; acethylation by p300, however, decreases the binding level of Myc with the promoter. Thus, p300 can participate in both activation and the inhibition of the hTERT promoter. The Myc/Max complex is acetylated differently, by p300 and GCN5, and is not acetylated by Tip60 in vitro, which means that the mechanisms of the action of these two transferases are different [59]. 

Histone deacetylation leads to a decrease in the expression of hTERT [60, 61]. The superexpression of histone deacetylases (HDAC1) leads to the suppression of the hTERT-promoter activity. HDAC1 binds to the telomerase promoter, inhibits telomerase activity, and interacts with Sp1 [62]. Histone deacetylases can interact with the hTERT promoter through the Mad1 protein, which binds to E-blocks [60, 62, 63]. 

Inhibiting histone deacetylation by Trichostatin A (TSA) leads to the activation of the hTERT expression; this effect depends on Sp1, but not on c-Myc. In the case of Sp1 superexpression, the effect of TSA is stronger; in the case of mutations in the Sp1-binding regions (but not with c-Myc), this effect does not occur [63]. Inhibiting histone deacetylation leads to an increase in the expression and activity of telomerase in normal cells-but not cancer cells (lung cancer cell lines)-that already have active telomerase [63, 64]. It is reasonable to suggest that, in the case of cancer, deacetylation is already switched off. 

### Cascade MAPK

The signaling cascade of Mitogen-activated protein kinase MAPK, which is an effector of the extracellular growth and stress signals, can regulate the transcriptional activity of many promoters by the direct phosphorylation of Sp1 or through other mechanisms. The MAPK-signaling Pathway is important for hTERT transcription regulation through the number of effectors which bind to the regions in its main promoter, including the transcription factors c-Myc, AP-1 and Ets [42]. The inhibition of the MAPK-cascade leads to a weakening of the phosphorylation of the estrogen receptor β (Erβ), to a decrease of the binding of ERβ to the hTERT promoters, and to the respective decrease in the level of hTERT expression [65].

### Oncogene Myc and its antipode Mad

E-blocks are located in positions -242 and -34 np of the hTERT gene, with whose oncoprotein c-Myc interacts, which is one of the main hTERT transcription activators [48, 66]. This protein is also a transcriptional activator of a number of promoters of other genes, and it is an inhibitor of the transcription of genes that are involved in the ceasing of cell growth [67].

It was shown that c-Myc expression suppression by anti-c-Myc antisense-oligonucleotides c-Myc, which was done on three leukaemia cell lines, also decreases telomerase activity [68]. c-Myc induces hTERT transcription and telomerase activity in normal cells of the epithelium mammary glands and primary human fibroblasts [69]. A high level of c-Myc leads to the activation of the hTERT promoter, but this effect disappears in the absence of E-blocks [66, 67, 70]. Blocking c-Myc bindings has a different effect in different cell lines. Thus, introducing mutations to the E-block (in position -242 np in the cells of lines C33A and ME180) leads to a fall in the hTERT promoter activity by 70%; however, in the cells of cell line SiHa, the same mutations have a very small effect. Introducing mutations into the E-block at position -34 np provokes a decrease of the hTERT promoter activity by 60% in cells ME180; however, hTERT promoter activity changes insignificantly in the cells C33A and SiHa [71]. c-Myc-induced hTERT promoter activation occurs quickly and independently of cell proliferation and protein synthesis [67, 70]. Heterodimers c-Myc/Max interact directly with the hTERT promoter [61, 66]. 

It is possible that N-Myc is another telomerase activator. This gene could be amplified in neurobalstoma at the same time as the hTERT promoter activation [Bibr R44][44]. Binding N-Myc with the hTERT promoter has already been shown [72].

The Mad protein is an antipode of c-Myc; it binds to the E-blocks as Mad/Max heterodimers and makes the hTERT promoter activity weaker. The suppression of hTERT promoter by Mad requires the activity of hitones deacylases. However, the inhibition of histone acetylases by TSA is not dependent on the presence of E-blocks in the hTERT promoter [60].

### Transcription factors Sp1and Sp3

Protein Sp1 regulates several specific promoters which initiate transcription by RNA-polymerase II in vertebrates. Sp1 binds to the sequence GGGGCGGGGC and similar sequences that are called GC-boxes. It regulates both hTERT and hTR promoters.

There is a set of regions for Sp1 binding in the hTERT that are required for promoter activity [46-48, 71, 73]. Similar clusters often exist in other promoters without the TATA-box, and they are required for its complete activation [74]. There are five known regions for Sp1 binding; they are located mainly between the promoter and E-blocks [46]. Two regions for Sp1 binding are identical for humans and mice [67]. It is very likely, judging by the active promoter regions, that sites for the Sp1 between E-blocks are involved in this regulation; introducing mutations into those sites leads to a decrease of promoter activity of different extents, depending on the mutated site and cell line [71]. In the case when those five binding regions are switched off, a 90% decrease of the hTERT promoter activity occurs, which means that Sp1 is absolutely necessary for its activity [60, 71]. Interestingly, that introduction of c-Myc activates the hTERT promoter in the presence of Sp1-binding regions; however, in the absence of those regions, the hTERT promoter activation by c-Myc is insignificant [71]. Two regions for Sp1 binding located in the region from -320 to -350np before the translation site and which are not yet well investigated and, possibly, two regions from -800 to -1000 np are probably involved in this regulation [47]. It is unlikely that other Sp1-binding sites that are further away from the transcription beginning site [47] are involved in hTERT transcription regulation.

Another ubiquitously expressed protein from the Sp protein family is protein Sp3, which often acts as a concurrent inhibitor of Sp1 [75]. Changing the ratio of those two proteins in favour of Sp3 leads to the inhibition of hTERT transcription [76]. 

Both Sp1 and Sp3 are necessary for repressing the hTERT promoter by histone deacetylases; they probably bind them to the promoter of TERT [77]. Apart from the above stated, Sp1 is also a transmitter of both activators and inhibitors of hTERT transcription (Fig 3).

**Fig. 4.**
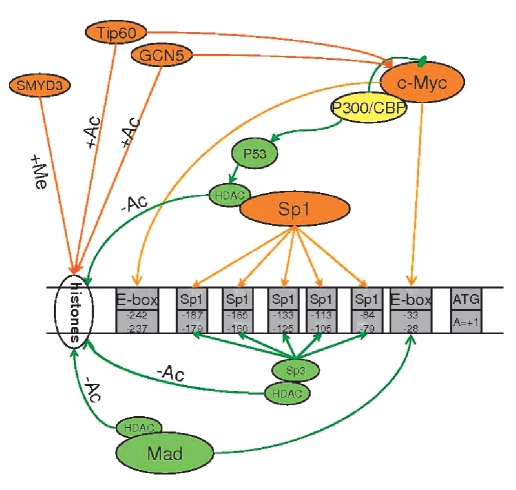
Methylation (+Me), Acetylation (+Ac) and De-acetylation (-Ac) of histons of hTERT promoter. The inhibitors of the hTERT promoter are green, activators are orange, and the effectors of double action are yellow. → marks the activation of the following player of the cascade, †is the inhibition of the following cascade player.

### Nuclear NF-kB 

NF-kB controls the expression and function of several genes involved in cancerogenesis [78] and, in particular c-Myc, which is a trans-activator of telomerase. When the protex Tax of the human Lymphotrophic virus type 1 (HTLV-I, Human T-lymphotropic virus I) activates NF-kB, the cascade increase of c-Myc and hTERT promoters occurs [79]. Chromatin immunoprecipitation showed that there is an increase in the binding of c-Myc and Sp1 to the hTERT promoter during its activation by NF-kB [80]. 

The expression of NF-kB and hTERT genes happens at the same time and increases at the early stages of stomach cancer formation [81]. The synthesis of hTERT and potential sites for the NF-kB binding in the hTERT promoter (regions -758--749 np and -664--654 np) at the same time allow us to suggest that NF-kB participates in telomerase activation [82]. The activation of the mTERT promoter (as a result of binding with NF-kB) was shown for mice [82].

NF-kB (p65 subunit) can interact with histone acetylases (HDAC1): because of this, it plays a role in the negative regulation of gene expression [83]. Histone deacetylases inhibit the hTERT promoter and bind to the promoter with the help of Sp1. NF-kB, in the case of binding with the promoter, can be used for binding HDAC1. 

### Transcription factors AP1 and AP2

The transcription factor AP1 (activator protein 1) participates in the processes of cell proliferation, differentiation, cancerogenesis and apoptosis; it is expressed both in cancer and normal cells. It is a heterodimer of Jun (c-Jun, JunB or JunD) and Fos (c-Fos, FosB, Fra-1 or Fra2) [84].

The super-expression of AP-1 leads to the suppression of hTERT transcription in the HeLa cell line. The combination of c-Fos and c-Jun or c-Fos and JunD decreases the activity of the hTERT promoter by 80% in experiments with a short time expression. The region of the hTERT promoter between nucleotides -2077 and -455 np participates in this process. JunD and c-Jun bind with both potential binding sites of AP-1 at positions -1732 and -795 np. Introducing mutations in the AP-1- binding sites at the hTERT promoter compensates AP-1-induced inhibition [84]. The correlation between the c-Fos expression, which is one of the AP-1 subunits, and the hTERT mRNA expression was shown [85].

The hTERT promoter also contains the potential region for the AP-2- binding [48, 86]. AP-2 binds with the region from -121 to 129 of the hTERT promoter. In rhabdomyosarcoma cells, the mutation in the AP-2 binding region leads to a decrease of promoter activity. At the same time, the super-expression of AP-2 does not lead to an increase in the hTERT promoter's activity [86].

### Onco-suppressors p53 and p73

Protein p53 regulates plenty of genes that play a role in the control of the cell cycle and oncogenesis (p21, MDM-2, Bax, c-Fos/Jun, pRB, 14-3-3σ, Bcl2 and others) [87-89]. p53 suppresses oncogenesis by switching on the mechanisms of cell cycle arrest and apoptosis as a response to different cell damages [87]. This protein is not active in more that half of human tumors [90]. Recovering the functional p53 in the case of cervical cancer, Burkitt's lymphoma, cancer of mammary glands, and pancreas leads to the inhibition of telomerase activity through the inhibition of the hTERT expression [91-94]. This effect appears several hours after the induction of p53, before the beginning of cell cycle abnormalities and apoptosis.

Mutations in the domains of p53, which are responsible for histones acetylases and co-repressor Sin3A, do not affect hTERT inhibition; even deacytilases participate in the inhibition of hTERT transcription by protein p53. p 53 mutations in the domain of DNA-binding, oligomerisation domain, or domain of transcription activation lead to the deactivation of p53 in relation to the telomerase. However, p53 does not bind with hTERT promoter in vivo, which means that its action on the promoter is non-direct. p53 can use proteins p21, E2F and proteins of group pRB [95].

Sp1 is necessary for the inhibition of p53 [92, 94]. Mutations in the regions of Sp1 binding inhibit the hTERT promoter activity by p53 [92]. Experiments on the cells of Drosophila Schneider SL2 have shown that the Sp1-ectopic expression-dependent hTERT-promoter activation is eliminated by wild-type p53. p53 interacts with Sp1 and blocks its binding to the hTERT promoter in vitro [94]. It is possible that p53 uses protein p21 as a messenger [95]: however, in previous similar experiments it was found that p21 is not a messenger between p53 and telomerase [94]. 

Protein p73 has an onco-supressing function similar to p53. Research on cells without p53 has shown that the super-expression of C-end isoforms p73 (α, β, γ, δ) leads to a decrease of hTERT-promoter activity. The supression of hTERT expression happens with the mediation of the endogenous p73 after the activation of E2F1 in cells. Mutations in the regions of Sp1 binding in the mane region of the hTERT-promoter rescue the repression of the hTERT-promoter by p73, which means that Sp1 acts as a messenger in this process. Apart from this, p73 binds Sp1, which proves the participation of Sp1 in the p73-dependent suppression of the hTERT expression [96].

### Proteins pRB, E2F, p21, and p16 

Proteins E2F can both suppress and activate oncotransformation on model systems [97]. The super expression of E2F-3 correlates with the worst prognosis in the case of prostate cancer, ovarian cancer, and nonsmall cell lung cancer; a high level of E2F-1 exists in case of lung, mammary, and pancreas cancer. A low expression of E2F-1 is obtained in case of a more aggressive illness during colon cancer and urinary bladder cancer. E2F-1, E2F-2 and E2F-3 are also regulated by protein pRB, but E2F-4 and E2F-5 are mainly regulated by proteins p107 and p130. E2F-1, E2F-2, and E2F-3 can bind Sp1, but E2F-4 and E2F-5 do not interact with it [98].

Protein E2F-1 binds to the telomerase promoter at twp specific regions (at the regions -251 and -175 np). Apart from this, there is a nonclassical E2F-1-binding region at from -67 to 61 np [99]. E2F-1 decreases the expression of hTERT m RNA and telomerase activity in the cell lines of squamous cell carcinoma [99, 100]. The ectopic expression of E2F-1, E2F-2, and E2F-3 leads to a decrease in the activity of the promoter of telomerase reverse transcriptase in cell lines HeLa, U2OS, and 273 with the mediation of Sp1; E2F-4 and E2F-5 do not inhibit telomerase. However, in the hTERT cells that are not transformed, the activation of endogenous telomerase occurs as an effect of E2F-1,2,3,4,5 [98].

Other results were obtained when researching [101] the cells of squamous cell carcinoma. It was shown that the activity of the hTERT promoter and telomerase activity are decreased only by the super-expression of pRB: however, the super-expression of E2F-1 restores the hTERT promoter acivity. In research on glioblastoma, a correlation between the expression of E2F1 and hTERT was found; patients with a low level of E2F-1 expression had a much better prognosis [102]. The ectopic expression of exogenous E2F-1 increases the activity of the hTERT promoter in cell lines Saos-2, HeLa, and U-251 MG [102]. The variation in the data could be related to the heterogeneity of the cell lines (for example, cell line HeLa has been cultured for decades in different laboratories, and could be very different depending on the source). Another reason for this could be the importance of not only the presence of E2F proteins for the inhibition activation of the hTERT promoter, by also of their posttranslational modifications and of modifications of pRB, which also participates in this cascade. 

E2F-1 could be one of the p53 messengers. The mutations of the noncanonical region for the binding of E2F-1 and super-expression of the mutant E2F-1, which binds to DNA but without the domains of transactivation and binding for the pRB, lead to the complete compensation of the p53 effect. The same effect occurs after the inhibition of proteins pRB, p107, and p130 [95]. 

The super expression of p21 and pRB completely suppresses the hTERT expression and stops the cell cycle in the cell line U-251 MG [102]. The appearance of protein p21, a cycline-dependent kinase inhibitor, leads to the accumulation of the hyperphosphorilated active form of pRB, p130, and p107. Those proteins bind to the proteins of the E2F family and transform them from activators of transcription to repressors [103]. Recovering the expression of pRB in pRB- and p53-negative cancer cells leads to the suppression of telomerase activity and stops the cell cycle [104]. The superexpression of the cyclin-dependent kinases cdk2 or cdk4, or of cycline D1 or E2F-1, leads to the restoration of pRB-supressed telomerase activity. For the functioning pRB as an inhibitor of the hTERT promoter, the phosphorilation of pRB is critically important [101]. The inhibition effect of pRB can be explained as binding to E2F-1 with further binding to the hTERT promoter [101] and particularly with bringing additional inhibitors, for example, histone deacrtylases. Also, protein pRB can disturb the binding of E2F-1 with the hTERT promoter. pRB and E2F-1 can also regulate an expression independently. 

Onco-suppressor protein p16 acts in the regulation of the pRB/E2F system. Its expression significantly decreases the level of telomerase activity in glioma cell lines. p16 inhibits the binding of Sp1 with its binding regions in the promoter [105]. It was shown [100] that p16 insignificantly inhibits telomerase in the cell lines of head and neck epidermoid cancer; in [101] it was shown that telomerase activity disappears completely in the cell line SSC25, which was transfected by the p16-containing vector. 

### Bcl2

Bcl2 is one of the apoptotic factors [106]. 

The superexpression of Bcl2 in human cancer cells with a low endogenous level of expression of this protein causes an increase in the level of telomerase activity. When the Bcl2 expression was switched off and activated after that of the cell line CTLL-2, telomerase activity also decreased and increased correspondingly and reversibly [107]. When researching the number of mammary gland samples, no correlation between Bcl2 expression and telomerase activity was found. Also, after the inhibition of the Bcl2 expression in the leukemia cell line HL-60, no changes in hTERT mRNA expression were obtained [109]. 

Possibly, the regulation of the expression of hTERT by the protein Bcl2 is not used or used very rarely during natural ontogenesis; the hTERT activation by Bcl2 that was found is not direct. Another explanation could be that this process is tissue-specific.

### Onco-supressor WT1 

Onco-supressor WT1 participates in the inhibition of telomerase activity [110]. In the hTERT promoter at the position -352 np, there is a WT1-binding site, the mutations in which can increase the activity of the hTERT promoter (it can increase it in cell line 293 but not in cell line HeLa). The superexpression of WT1 suppresses the expression of hTERT, mRNA, and telomerase activity in cells 293 [110]. Because gene WT1 is expressed in certain types of cells during differentiation (kidney, reproductive organs, spleen) [111], the role of WT1 in the telomerase inhibition is probably tissue-specific. 

### Myeloid cell-specific protein MZF-2 

In the hTERT promoter, there are four possible regions for the binding of transcription factor MZF-2 at the positions -687, -619, -543 and -514. They are responsible for suppressing the activity of the hTERT promoter, and MZF-2 specifically binds with those regions. The superexpression of MZF-2 suppresses the activity of the hTERT promoter [112]. 

MZF-2 is expressed in cancer telomerase-positive cell lines, and it seems that this protein does not play a major role in telomerase inhibition [112].

### Regulator protein of the USF group

The hTERT promoter contains E-blocks with which not only dimmers Myc/Max and Mad/Max can bind, but regulator factors USF as well. In the model system with a reporter vector, the expression of USF1 or USF2 inhibits the activity of the promoter. These proteins do not interact with c-Myc or Mad and do not influence their expression in the cell: however, they directly bind with E-blocks in the hTERT promoter. Analyzing the clinical cancer and normal samples from the mouth has shown that the level of expression of USF1 and USF2 is lower in the cancer samples, but the hTERT expression and telomerase activity is higher in the cancer samples [113].

According to other data, USF1 and USF2 as heterodimers act with both the binding regions in the hTERT promoter and do not affect the hTERT transcription in hTERT-negative somatic cells. In hTERT-expressing cells, these proteins activate transcription and participate in the appearance and support of the cell immortality [114].

### Transcription factors ETS

ETS is a family of transcription factors; these proteins contain a conservative DNA-binding domain which specifically acts with GGA(A/T) sequences. 

MAP-kinases can phosphorilate proteins ETS1 and ETS2 after activation by the Epidermal Growth Factor (EGF) and its analogue HER2/Neu. The phosphorilated form of ETS is active in transcription. Culturing cancer cells A-431, ME180, and MCF-7 c EGF makes the hTERT promoter stronger. The effect of EGF is compensated after the addition of the MAP-kinase inhibitor or after removing two supposedly closely located regions of ETS binding factors with the promoter in the region c from -22 to -14 np. EGF can lead to the phosphorilation of c-Myc, and, as a result of this, c-Myc activates transcription: however, mutations in the regions for c-Myc binding in the hTERT promoter do not affect the ability of EGF for transcription activation [115]. ETS interacts with the hTERT promoter DNA at the -36 np-position with activation, but it interacts at the -293-np position with the inhibition of hTERT expression, forming the complex Ets-Id2-DNA (Id is a family of regulator proteins of cell growth and differentiation inhibitors) [116]. The superexpression of ETS1 and ETS2 leads to a decrease and the superexpression of Id2 leads to an increase of telomerase activity in the K562 cell line [117].

Onco-protein HER2/Neu activates the transcription of hTERT; it uses the transcription factor ER81, a member of the ETS family, as a messenger. An expression of ER81 only or HER2/Neu in the cell line BJ does not stimulate an expression of hTERT mRNA. An expression of both of these genes induces the transcription of hTERT, mRNA, and telomerase activity. In the model system, the expression of ER81 and HER2/Neu increases the promoter activity 3 and 9 times, correspondingly, and their combined expression increases the promoter activity 37 times (in the cell line 293T). By now, five possible regions for binding have been found, and for two of those regions (at the positions +211 - +214 np and +313 - +316 np) the ability to bind with ER81 has been shown. Mutations of only those two regions lead to a cooperative decrease of the activation of the hTERT promoter by ER81 and HER2/Neu [118]. ERK MAP-kinases are mediators between HER2/Neu and ER81. It was also shown that Ras and Raf, which are regulators of ERK MAP kinases, stimulate the transcription of hTERT [118]. 

PEA3 and ERM, two other proteins of the ETS group that are from the same subfamily as ER81, in collaboration with HER2/Neu, activate the hTERT promoter, but to a lower degree than ER81. On the other hand, four transcriptional factors of the ETS group from other subfamilies (Elk1, Sap1a, Elf1, and ER71) practically do not activate the hTERT promoter [118].

### Signaling the pathway of receptor Ck 

A disturbance of the signaling pathway of the cholesterol-specific Ck receptor was obtained in leukemia patients, in the leukemia cell lines, and in the Central Neural System Cancer.

The Ck active receptor decreases the expression of hTERT mRNA by inhibiting proteinkinase C. Proteinkinase C activates the transcription of PPARγ (peroxisome proliferated receptor γ), which inhibits the expression of c-Myc and hTERT and decreases telomerase activity. Also, PPARγ can interact with transcription factor Sp1, which is an activator of the transcription of hTERT. Apart from this, PPARγ is an antagonist of NF-kB, which is also an activator of the transcription of hTERT [119].

### Steroid hormones

In many cases the probability of oncogenesis can be increased as a result of disturbances in the hormone-mediated regulation of gene expression. Several hormones that participate in cancerogenesis could affect the expression of hTERT.

### Estrogens. 

Estrogen (17β-estradiol) activates hTERT transcription in hormone-sensitive tissues. After the treatment of mammary gland cancer cells or normal cells of ovarian epithelium by estrogen, the level of hTERT, mRNA, and telomerase activity increases within a couple of hours [120, 121]. An analysis of the hTERT promoter has shown that there are two regions for esrogen-receptor binding [122]. The region for binding the estrogen receptor at the position -2754 np increases the activity of the promoter by five times as an effect of the hormone. After removing this region, the activation hTERT promoter by estrogen decreases by 70%. The second region, at the position -949 np, probably works in cooperation with the Sp1 site that is located nearby [123]. It was shown using the foot printing approach that protection of the region at -949 np is effected in the presence of estrogen. Mutations of this region strongly decrease the activation of the hTERT promoter by estrogen in the reperter construction [121]. In another work it was found that the estrogen receptor is bound only with the -2754-np region, but not with the -949-np region; it was also found that removing the -949-np region from the hTERT promoter does not affect the activity of the promoter [120]. Both receptors α and β bind to the hTERT promoter. The low telomerase activity in the mammary cancer correlates with the absence of the estrogen receptor β [33]. The activation of the hTERT promoter in the cell line NIH3T3 depends on the presence of the α estrogen receptor, but not on β [121]. 

The scheme of the multi-level activation of telomerase is achieved during estrogen regulation. Estrogen activates telomerase not only as a direct regulator, but also through the induction of c-Myc, which is another activator of the hTERT promoter [120]. 

Estrogen also activates hTERT expression through the PI3K/Akt/NF-kB cascade. Estrogen also induces the phosphorilation of hTERT, binding proteins 14-3-3 and NF-kB with the hTERT, and the Akt-kinase mediated accumulation of hTERT in the nuclei [124]. 

### Androgens 

Normal prostate tissues and epithelial cell lines usually do not have telomerase activity in the presence of androgens, but the absence of androgens leads to telomerase activation in normal rat prostate tissues and does not produce significant changes of telomerase activity in the heart, kidney, liver, and lung tissues [125]. However, most types of prostate cancer have strongly expressed telomerase activity at the normal level of androgens. In the cell lines of the prostate cancer, telomerase activity is suppressed in the absence of androgens [126]. Antibody staining has shown a significant suppression of telomerase in a set of clinical samples of a prostate without androgens [127]. 

In the cell lines of the mammary gland and uterus cancer, progesterone increases the mRNA of the Telomerase Reverse Transcriptase within three hours of treatment. After 12 hours, its amount peaks and starts to decrease; after 48 hours progesterone opposes estrogen and inhibits the estrogen-induced expression of hTERT mRNA. The activating effect of progesterone happens through a cascade of MAP-kinases, and inhibition happens by p21 [128]. Combining estrogen and progesterone (which models a decrease in the risk of mammary gland cancer during pregnancy) leads to the suppression of telomerase activity and increases the expression of its inhibitors p53 and p21 in the cell line of the mammary gland epithelium 76N TERT [129].

During the growth of normal and androgen-independent prostate cell lines in the presence of dihydrotestosterone, no changes in telomerase activity were obtained. In the case of an androgen-dependent cell line of LNCaP prostate cancer, the absence of androgen in the media leads to a decrease in telomerase activity. Dihydrotestosterone activates telomerase activity in the G1-phase of the cell cycle. However, there was no increase of promoter activity obtained in the experiments with reporter construction [130, 131]. The action of androgen is non-direct, and it is consistent with the absence of elements of the response to androgen in the promoter of hTERT [126]. 

### The Viral Regulation of hTERT Expression

#### Human papillomavirus

Human papillomaviruses (HPV) are divided into 3 groups: non-oncogenic, low risk, and high risk by the probability of neoplastic transformation of contaminated cells. Proteins E6 and E7 of HPV from the high-risk group participate in oncogenesis by inactivating cancer suppressors p53 (E6, together with E6AP ubiquitin protein ligase), pRB and pRB-associated proteins, p130 and p107 (E7), and some other proteins [132]. 

After the transfection of telomerase-negative cells (primary keratinocytes) by genes of E6 and E7, only E6 (and not E7) activates telomerase [133, 134]. After the transfection of cells by both genes (E6 + E7), the data is contradictory: the activation of telomerase can be the same as at transfection only with genes E6 [134], somewhat lower [133], or somwhat higher [73]. In the cell line of cervical cancer C33A (with active telomerase, but without HPV), an expression of E6 activates an expression of hTERT by 3 times and E7 by 1.5 times [73]. In the line of mammary gland epithelium, E6 activates telomerase almost immediately but E7 accelerates the process of telomerase activity appearance in the cell population gradually (high telomerase activity occurs after 20-25 passages with the expression of E7) [135]. Shortly, E6 is a direct telomerase activator, and the possible activation of telomerase by the protein E7 is a mediated and weak effect.

E6 activates telomerase transcription through the promoter region from -260 np until the site of translation initiation (from -15 to -266 np [134], -258 np [73]), in which there are 2 regions of c-Myc binding; when any of these two regions are removed, the activity falls by 60% [134]. E6 is coprecipitated with c-Myc by the immunoprecipitation method [136]. No changes in the c-Myc expression level after the transfection of cells by the gene of E6 appeared. After the superexpression of the gene that encodes Max (protein-antagonist of c-Myc), the E6-mediated suppression of hTERT occurs [134]. According to the data of other authors, introducing mutations into both regions of c-Myc binding leads only to a minor decrease of the promoter's activity. However, mutations in Sp1-binding sites lead to a decrease of E6-induced promoter activity by 50%. Mutations in the regions of c-Myc and Sp1 binding at the same time lead to the almost complete disappearance of the telomerase transcription activation by protein E6 [73].

In keratinocytes and mammary gland epithelium cells, E6 from the HPV of a high-risk group (HPV 16, HPV 18, HPV 31, and HPV 54) has a strong telomerase activation effect; however, in the case of HPV from the low-risk group (HPV 11, APV 6), hTERT transcription activation is not strong [73]. Only the protein E6 of papillomaviruses from the high-risk group binds to the minimal promoter of hTERT (-300 to +1 n.p.) [136]. The activation of telomerase by the protein E6 is cell-type specific. The transfection of cells by gene E6 (which, in the case of the cell of uterine cervix epithelium, leads to the telomerase activation in the case of foreskin fibroblasts [137] or cells IMR90 [69]) does not give a similar effect.

Protein E2 HPV can inhibit the hTERT promoter. This protein can bind with the hTERT promoter. For the promoter inhibition, the interaction with Sp1 (the binding regions which are located between E-blocks) is important. E2 can inhibit the growth of HPV-infected cells and leads HeLa cells to apoptosis [138]. 

#### Hepatitis B virus

Protein X of the Hepatitis B virus (HBV) is a transactivator whose targets are the genes c-Myc, AP1, AP2, and NF-kB [19], which are activators of telomerase.

The frequency of telomerase activity appearance increases from the normal tissue to the cancer tissue: in 79% of cancer cases, 24% of cirrhosis, and 8 % of chronic hepatitis [32]; in 85,2% of cancer cases, 45,9% of cirrhosis, 25% of chronic hepatitis, and 15,7% of normal tissues [41]. 

After the transfection of cell lines by the gene of the protein X HB, an increase in the hTERT mRNA was shown (cell lines of hepatic carcinoma and cholangiocarcinoma) [139]. Western Blot has demonstrated that the amount of Telomerase Reversed Transcriptase increases in the hepatoma cell line after the superexpression of the protein X HBV [140].

A slight increase in c-Myc, together with hTERT, after the superexpression of protein X in the hepatoma cell line was also found [140]. Because c-Myc is activated by protein X and activates the expression of hTERT, it is, possibly, one of the mediators in the activation of telomerase after contamination by HBV. However, the relationship between the hTERT mRNa expression and the level of c-Myc was not found by in situ hybridisation in clinical hepatic cancer samles. Mutations were found in the regions of the c-Myc binding to the hTERT in clinical samples of hepatocellular carcinoma [141]. 

The regions of possible binding of the nuclear hepatocellular factors HNF-3b and HNF-5 were found in the promoters of TERT in both humans and mice. They are conservative, but their functional significance has still not been defined [67]. 

#### Herpes viruses (Epstein-Barr virus, Kaposi's sarcoma) 

Both herpes viruses, which are oncogenic for human, can participate in the regulation of the transcription of hTERT. 

The Epstein-Barr virus (EBV) is a causative agent of infectious mononucleosis and is related to cancerogenesis, for example, Burkitt lymphoma [19, 142]. The latent membrane protein 1 (LMP1) of EBV induces the specific binding of hTERT with the p65 subunit of NF-kB and the transfer of both proteins from the cytoplasm to the nuclei [143]. Another mechanism of hTERT expression activation by the protein LMP1 occurs through its c-Myc [144]. The latent membrane protein 2A LMP 2A suppresses the transcriptional activity of the hTERT gene [145].

Herpes virus type 8 is identified as a causative agent of multiple pigmented sarcoma (Kaposi) [19]. The nuclear antigen LANA of this virus is an activator of hTERT transcription. This protein can bind with Sp1, and the activation of the telomerase promoter probably occurs as a result of this interaction [146]. 

#### T-lymphotropic viruses 

T-lymphotropic viruses of the 1st and 2nd types (HTLV-I and HTLV-II) activate telomerase [80, 147]. 

All adult patients with acute or chronic T-cell leukemia demonstrate a high activity of telomerase; however, nonsymptomatic HTLV-I carriers show telomerase activity only in 29% of cases. Two out of seven patients with active telomerase transformed to the acute form in one month [148]. However, the lack of data (there were only 24 patients) does not allow one to draw a definite conclusion about a direct relationship between the increase of telomerase activity and disease progression. Telomerase activity is high in both HTLV-I transformed cells and in lymphocytes from leukemia/lymphoma patients when compared with nontransformed or normal cells [149].

In the regulation of hTERT transcription, the protein Tax of the HTLV-I virus participates; even this protein is oncogene: it can suppress the expression of the gene hTERT twice in three days [80, 150] after the stimulation of cell division by phytohaemagglutinin. In the absence of phytohaemagglutinin and in the presence of a Tax, there is an increase of telomerase activity by 25% in the same time span. The activation of the expression of NF-kB happens during this process, which activates the hTERT promoter [80]. The suppression of the hTERT promoter activity happens as a result of the competition between Tax and c-Myc for the canonic region of c-Myc binding in the hTERT promoter [150]. 

#### SV 40 and adenoviruses. 

Neither SV 40 nor adenoviruses (AV) are related to the ethiology of natural types of human cancers, but in the model systems they can act in the transformation.

AV are not oncogenic for human, and gene therapy uses them for the creation of genetic material delivery vectors. However, the expression of adenoviral protein E1A is enough for the immortalisation of rodent primary cells, and, in the presence of a second oncogene E2A or ras, their transformation is possible. Protein E1A 243R and, particullary, second exon E1A, activates the promoters of hTERT and hTR. This activation could be suppressed by the repressor CtBP (C terminal binding protein), which does not influence the basic level of hTERT expression by its own. The activation of hTERT by protein E1A probably occurs through the regions of Sp1 binding [151]. These data show that it is very important to be extremely careful with AV and with the nonocogenicity-safety of their use in human therapy.

Virus SV 40, which has long been a suspected oncogene, is not considered dangerous for humans [152]. In human cell lines, big and small SV40 antigens can lead to transformation [153]. There is data that virus SV40T accelerates the appearance of telomerase activity in human mammary gland epithelial cells. But this activity is dependent on the number of passages, which means that telomerase activation may happen indirectly and as a result of an increase in the frequency of some random event that happens as a result of cell metabolism abnormality [135].

### The effect of hypoxia on the hTERT expression 

The regions of cancer hypoxia are characterized by resistance to therapy, genetic instability, and by increasing malignancy. Hypoxia can lead to the intensification of telomerase activity, for example, in the cell lines of cervical cancer [154]. In its main part, the hTERT promoter contains two regions of a hypoxia responsible element (HRE) for binding hypoxia-inducible factor-1 (hypoxia-inducible factor-1 and HIF-1) in the regions -242 and -26 np. It was found that these regions are necessary for the activation of hTERT by HIF-1 [155]. The incubation of cancer cell lines in the lack of oxygen leads to the assembling of a transcriptional complex that includes HIF-1, p300/CBP, RNA-polymerase II, and TFIIB on the hTERT promoter in the region of HRE. The superexpression of HIF-1 in the ovarian cell line leads to the growth of hTERT promoter activity by almost two times. During the growth of a cancer cell line in the condition of hypoxia without the superexpression of HIF-1, the redistribution of splice forms of hTERT mRNA takes place (see in detail in the section "Regulation of hTERT splicing") with a very insignificant increase in the total hTERT mRNA [156]. 

### Posttranscriptional regulation of hTERT

Cancer needs the support of the telomeres′ length, and the activity of telomerase that supports the telomeres′ length may not be in correlation with the transcription of hTR and/or hTERT mRNA [32]. 

### Regulation of hTERT splicing 

**Fig. 5.**
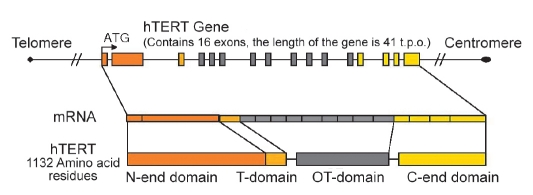
Gene of telomerase reversed transcriptase. Regions that encode N- and C- ends, reverse-transcription (RT), and domains typical for telomerases (T) are shown [48].

The telomerase reversed transcriptase gene consists of 16 exons and makes up ~37,000 base pairs of genomic DNA, in which introns make up ~33,000 base pairs and ~4,000 are related to the transcript [48] (Fig. 5). 

### Natural splicing of hTERT mRNA

**Fig. 6.**
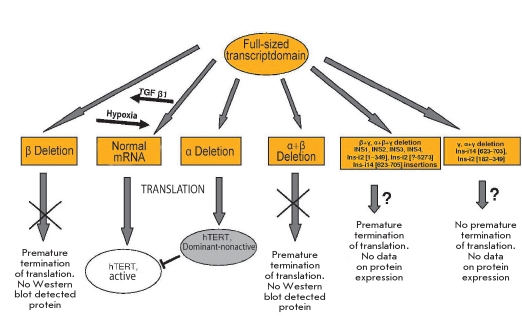
Different variants of splicing of the hTERT mRNA.

Only full-sized hTERT mRNA provides telomerase activity. Thirteen alternative splicing variants of hTERT mRNA are known [48, 157-159]. Isoform with α-deletion (the deletion of 36 nucleotides in the reverse-transcription domain) act, after superexpression, as the dominant inhibitors of telomerase activity [160, 161]. This variant of hTERT mRNA is translated, and the appearing protein can be included in the telomerase dimer complex (Fig. 6).

Deletions β and α+β do not lead to the formation of the active telomerase, but neither do they inhibit it [161]. However, after the treatment of immortalized cell lines with the TGF β1 (transforming growth factor β1), the β-variant of hTERT mRNA that is formed as a result of alternative splicing and telomerase activity drops [162]. The opposite situation could exist at the regions of cancer hypoxia: the redistribution from the spliced β-variant to the active transcript [156] (Fig. 6).

Telomerase activity in the cells of osteosarcoma, which express only full-sized mRNA, is higher than in the case of cells with a set of different splicing forms of hTERT mRNA [54]. Telomerase activity increases in the stomach adenocarcinoma tissue, the total amount of hTERT mRNa is increased compared to the surrounding tissue, and the ratio of the amount of α, β and α+β forms is the same, which indicates the absence of regulation of the splicing of hTERT [163].

The variant with the deletion γ is expressed at a low degree and does not affect telomerase activity in the cell lines that were derived from hepatic cancer [159]. Deletions α+β, β and 4 insertions INS 1-4 [48, 157] cause the early termination of hTERT translation [161] similarly to variants with deletions β+γ, α+β+γ [159]. Part of the transcripts that were found recently [158] have still not been investigated in relation to telomerase activity. 

Possibly, splicing telomerase regulation is tissue-specific. An analysis of embryonic tissue has shown that telomerase activity in the heart and liver correlates with the hTERT gene expression: in a kidney, this activity disappears on the 15th weak of development, and hTERT transcripts could be found at the 21st week [164].

### Artificial splicing 

Intron of the first group from Tetrahymena can induce new RNA, which substitutes hTERT mRNA with a high accuracy and specificity as a result of trance splicing [165]. In the cell line of prostate cancer 2'-O-methyl-RNA phosphothioate oligonucleotides, which are specific to the splicing region between the 5th intron and 6th exon in hTERT pre-mRNA, there is a decrease in the amount of full-size transcripts and, at the same time, an increase in the amount of alternatively spliced transcripts, which leads to a decrease in telomerase activity. The growth rate decreases in this process, and cells start apoptosis within two days [166].

### Cellular localization 

Even telomerase activity needs the localization of telomerase in the nuclei; near the telomeres, a high activity of telomerase in vitro occurs in the cytoplasmatic extract, but not in the nuclear extract [167]. However, in vivo GFP-containing telomerase is located in the nuclei [168].

When the binding of NF-kB (p65 subunit) with the protein hTERT occurs, the tumor necrosis factor α (TNF α) induces the transfer of NF-kB-bound hTERT from the cytoplasm to the nuclei [169]. 

Protein 14-3-3, which is responsible for nuclear localization, binds with telomerase. Dominant-negative 14-3-3 directs hTERT, which is normally localized in the nuclei, into the cytoplasm. Mutant hTERT, which is incapable of binding with 14-3-3, localizes in the cytoplasm. 14-3-3 disturbs protein CRM1 binding with the c NES-motif (nuclear export signal). Inhibiting the CRM1/exportine1-pathway of the nuclear transport, as well as damaging the NES-motif, leads to a decrease in the localization of hTERT in the cytoplasm [170].

During most of the cell cycle, hTERT is not localized in the nucleoli, Cajal bodies, or telomeres. At the S-phase of the cell cycle, hTERT moves to the nucleoli, then to the Cajal bodies, and then to the telomeres [15, 16]. 

### The phosphorilation and dephosphorilation of hTERT

The phosphorilation of the telomerase reversed transcriptase by the proteinkinase Cα (PKCα) is required for the telomerase activity in mammary-gland cancer cells [171]. Another proteinkinase from this group, proteinkinase Cz (PKC zeta), controls the telomerase activity in the cell of the cancer of the nasopharynx without any effect on hTERT expression. Switching off the PKC-activator zeta Cdc42/Rac1 leads to a decrease of telomerase activity [172] (Fig. 6).

**Fig. 7.**
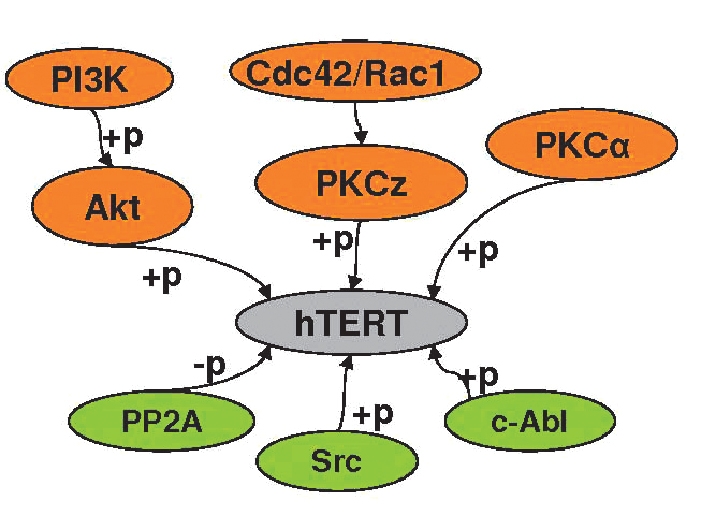
Regulating the activity of the protein hTERT by phosphorilation-dephosphorilation. Activators are shown in orange, inhibitors are shown in green. "+p" means phosphorilation, and "-p" means dephosphorilation.

Phosphatase PP2A inhibits telomerase activity in mammary-gland cancer cells [173] (Fig. 7).

The kinase Akt increases telomerase activity by phosphorilation hTERT in the melanoma cell line [174]. The dominant-negative mutant Akt-kinase significantly decreases the level of telomerase activity in the endothelial cells. Also, it suppresses telomerase activity by inhibiting kinase PI3K (phosphoinositol 3-kinase), which phosphorilates and activates Akt-kinase [175] (Fig. 7).

Phosphorilation not only directly affects the activity of hTERT, but it also affects the transcription of this gene. The treatment of cells by the PI3K inhibitor or the expression of the dominant-negative Akt-kinase in the cells makes the estrogen-activated hTERT activity weaker in the human ovarian carcinoma cell lines [124].

Two-strand DNA gaps, activated Tyrosin-kinase c-Abl, bind and phosphorilate hTERT, which inhibits its activity. The irradiation of cells by ionizing radiation induces the phosphorilation of hTERT by the c-Abl-dependent mechanism [176].

Under oxidative stress conditions, GTP-ase Ran provides the export of hTERT. hTERT-phosphorilation by kinase Scr is required for this export. [177]. The superexpression of phosphates Shp-2 blocks this mechanism of exporting hTERT [178].

### The regulation of telomerase RNA

In adult human tissues, a high level of telomerase RNA was found in primary spermatocytes and in the Sertoli cells, a middle level of expression was found in lymphatic follicles, and a low level of expression was obtained in the epithelium; hTR expression is absent in the neuronal system and in mesenchyme-derived tissues [179]. A significant level of expression was found in the small intestine, thymus, kidneys, and prostate. A low level of hTR expression is obtained in the brain, liver, stomach, pancreas, lungs, and heart [180].

In telomerase-positive samples of tumor tissues, a strong expression of hTR was demonstrated, but only half of telomerase-negative soft tissue sarcomas expresses hTR to a different degree. In telomerase-negative tumors, there is no relation between the expression of hTR and the proliferating status, telomeres length, and expression of hTERT [Bibr R181][181]. The high expression of hTR is not related to the telomerase activity, in lung cancer, for instance [30]. 

Using in situ hybridization, it was demonstrated that, in the case of Barrett's esophagus and of early stages of esophageal dysplasia, hTR is absent or expressed at a middle level; however, the effectiveness of hTR expression is high at late developmental stages of dysplasia or cancer [182]. By using RT-PCR (reverse transcription and PCR), hTR is detected in 90-100% of the samples of lung cancer, both in cancer and normal tissues [30, 183]. Using in situ hybridization, hTR is detected only in 26% of cancer tissues that are defined by histological analysis; for some samples, a difference between cancer and normal tissues was obtained. The same approach detected an expression of hTR in 41% of squamous cell carcinoma, 13% and 17% of adenocarcinoma of mammary gland and ovary, 43% of cancer, and 40% of cervix uteri precancerous lesions [34]. Unfortunately, the further fate of patients with precancerous lesions is unknown, so it is impossible to discuss the expression of hTR as a marker of oncogenic process development. In order to use hTR expression as a cancer marker, it is necessary to do a precise quantitative analysis, unlike the difference in expression of hTERT or determination of telomerase activity.

An analysis of nueroblastoma samples has shown high or middle level hTR expression in 9 out of 12 samples of the middle stages of cancer and only in 2 out of 8 samples of the early stages. The illness did not progress in patients with a low level or without the expression of hTR. In case of eight samples of skin cancer taken from seven patients with a middle or high level of hTR expression, the development of diseases was not good. 

It should be mentioned that, in late stages of diseases with large metastases (four samples), the expression of hTR was weak. The expression of hTR in the ganglyoneuroblastomas and gangloneuromas is located only in neural cells, and it is absent in lemmocytes. Therefore, hTR seems to be a good prognostic factor in non-metastasis neuroblastomas [184].

For Williams cancer, hTR (but not hTERT) is a predictive factor of further development. In 30% of patients with the highest expression of hTR (quantitation was done by Real-Time PCR), the probability of relapse was twice as high as in patients with the lowest level of hTR expression [185]. Also, the high level of expression of hTR correlates with the bad prognosis in patients with lyposarcoma [42].

Increasing hTR expression does not always correlate with the appearance of telomerase activity [30]. hTR inhibits the proteinkinase of the ATR checkpoint. Suppressing the level of hTR expression stops the cell cycle at the G1- and G2- phases as a result of the activation of p53 and of the proteinkinase of CHK1 checkpoints. This effect is not dependent on telomerase activity. Increasing the hTR expression as a response to ultraviolet irradiation stops the activation of p53 and CHK1 as a result of the inhibition of ATR activity; because of this, it makes the cell response to the DNA damage weaker and allows cells to pass the checkpoint G2/M. No interaction between hTR and ATM was found, and the mechanism of this inhibition is still unknown [186].

### Regulation of transcription of telomerase RNA 

hTR promoter contains CCAAT- and TATA-blocks near the region of the transcription start and several binding sites for the receptors of glucocorticoids, progesterone, androgen, and transcription factors AP1 and ETS [187]. The minimal region of the human telomerase RNA promoter is from -272 to -42 np prior to the start of transcription. The activity of the promoter is maximal when the region before -463 np is used; if the size of the region used is larger, the level of transcription decreases [187]. 

Investigating the methylation status of the hTR promoter has shown that three out of eight telomerase-positive cell lines and both telomerase-negative cell lines are hypermethylated; however, at the same time, no methylation of the hTR promoter was obtained either in cancer or in normal tissues. So, it is very likely that the methylation of the promoter is not related to the regulation of the hTR expression [181]. 

**Fig. 8.**
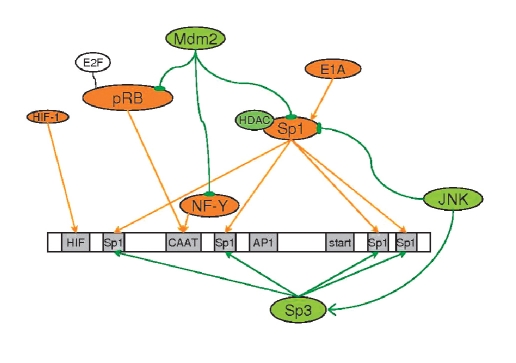
Scheme of the effect of transactivators of hTR promoter. Inhibitors of the hTR promoter are green, activators are orange, and cofactors are colorless. → means activation of the following cascade player, †means inhibition of the following cascade player, and the absence of an arrow means co-action.

hTR promoter contains four regions for the binding of proteins Sp1/Sp3 (Fig. 8). Sp1-binding activates the promoter, but Sp3 inhibits it [188]. Mutation analysis has shown that the region before the CAACT-block is required for the activation of the hTR promoter, as well as the fact that three regions after CAACT are responsible for promoter inhibition. The region immediately after CAACT has an inhibition effect at the strongest level but the lowest affinity Sp1. This can be explained because it is close to the region of the binding of transcription factor NF-Y. Two regions just after the start of transcription are under complicated regulation; the introduction of mutations into both regions results in a strong activation effect that is not just the sum of the effects of single mutations [189]. On the other hand, research [151] has shown that the introduction of mutations into four regions of Sp1-binding at the same time does not affect the base level of hTR promoter activity, although it disturbs its transregulation.

The transcription factor NF-Y, which is capable of recruiting several components of the RNA-polymerase II complex to the promoter [190], is the main activator of the telomerase-reversed transcriptase promoter. NF-Y binds to the region CCAAT of the hTR promoter, and the disturbances of this binding lead to the almost complete disappearance of promoter activity [189]. 

pRB is also an activator of the hTR promoter. The CAACT region is required for its action. The activation of the hTR promoter by the pRB protein decreases in case of mutant forms of pRB, which are not able to bind E2F, and it disappears in the case of the mutant δ657 form, which is not able to bind E2F or activate gene transcription [188]. 

Mytogene-activated proteinkinase kinase 1 - (MEKK1)/c-Jun-NH(2)-kinase (JNK) suppresses the expression of hTR. The transfection of the permanently active kinase domain of MEKK1, the main MAP3K in the JNK-pathway, leads to the strong inhibition of the hTR promoter in some cancer cell lines. The suppression of the hTR promoter by kinase MEKK1 could be blocked by the SP600125 inhibitor of JNK. The effect of hTR-promoter inhibition using kinase MEKK1 can be intensified by the co-expression of wild types of JNK, but not by the co-expression of the mutant form of JNK, which cannot be phosphorylated. The cotransfection of Sp3 and MEKK1 gives an additive effect of hTR inhibition. 

According to immunoprecipitation data, treating cells with SP600125 leads to a change in the ratio of Sp1/Sp3 on the promoter, increasing Sp1. Thus, this kinase helps change the Sp1/Sp3 balance on the promoter, increasing Sp3 without changes in the level of expression of Sp1 and Sp3 or the inhibition of the hTR promoter [191]. 

Ubiquitin lygase Mdm2 decreases the stability of p53 and regulates the pRb/E2F complex [192]. Mdm2 reacts with Sp1 in vitro and in vivo and inhibits the transcativation of Sp1-activated promoters. Mdm2 interacts with the promoter of hTR in vivo and inhibits it; however, regions for Sp1 binding do not act in this process. Mdm2 suppresses activation by pRb, NF-Y, and Sp1. Mdm2 and pRb, as well as NF-Y, can interact with the complex of RNA-polymerase II and, as a result, they can affect the expression of hTR [190].

The telomerase RNA gene contains the HRE region, and HIF-1 binds to this region. Superexpression HIF-1 in the cancer cells leads to an almost twofold growth in the activity of the hTR promoter in 6 h of incubation in hypoxia conditions, and then to a decrease back to the normal level in 4 hours. The transcription complex (including HIF-1, p300, RNA-polymerase II, and TFIIB), which was assembled on the hTR promoter in the cancer cell line in hypoxia conditions, was shown [156]. 

Adenoviral protein E1A increases the expression of the reporter vector under the control of the hTR promoter by 2.5 times. This activation likely occurs through the regions of Sp1-binding, because their mutations lead to the disappearance of the E1A effect. The activation of the hTR promoter by the E1A protein is also inhibited by the protein CtBP, but it does not influence the basic level of the hTR expression [151]

### The post-transcriptional regulation of telomerase RNA

TR is accumulated in the Cajal bodies in the cancer cell lines, but not in the cell lines of the normal cells. It is possible to provoke the accumulation of hTR in the Cajal bodies by the expression of the hTERT in the cells [193]. It is hTERT that is the most important factor of the hTR localization in both the Cajal bodies and on the telomeres [194]. The previous 3'-end of the hTR processing is also required for accumulation in the Cajal bodies [195]. 

The stability of telomerase RNA in cells can increase at oncogenesis. Upon expression of hTERT in the hTERT-negative cells, the period of hTR half-decay increases by 1.6 times. This may happen due to binding and stabilization by the TR catalic subunit [196]. 

### Control of telomerase access to the telomers 

**Fig. 9.**
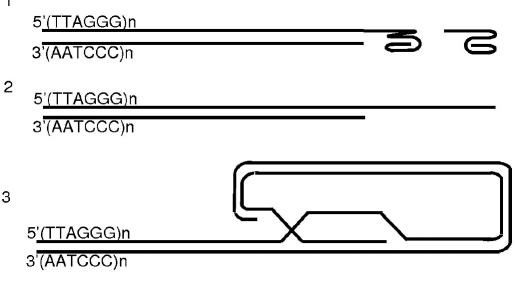
Possible organization of telomere ends: (1) examples of G-quadruduplexes, (2) protracting 3'-end (substrate for telomerase), and (3) T-loop.

Telomers have a projecting 3'-end which can form several structures: T-loop [197] and G-quadruplexes [198, 199] (Fig. 9). In the T-loop, the projecting 3'-end is a joint with the internal region of the telomere and is stabilized by proteins [197]. G- are formed by the projecting 3'-end because of the formation of Hughstein's pairs [198, 199]. In humans, six proteins (TRF1, TRF2, hRap1, TIN2, TPP1, and POT1) form a Shelterin complex that is a permanent component of human telomeres [200]. 

Changing the level of expression of the Shelterin complex strongly affects the telomerase length. For instance, inhibiting TRF1 leads to the telomeres lengthening in human cancer cells, and its superexpression leads to the shortening of telomeres without any changing of the telomerase activity in vitro. Decreasing the amount of protein TIN2 or the superexpression of its mutant alleles, which disturbs TIN2 binding to TRF1 and TRF2, leads to telomeres elongation. The superexpression of TRF2 causes telomere shortening; this happens not only because of the in vivo inhibition of telomerase, but also because of the increase in the velocity of the shortening. Suppressing TPP1 by RNA interference or disturbing the TPP1-POT1 binding also leads to telomere elongation, with the following loss of the protein POT1 by the telomeres [200]. According to the data of other authors, TPP1 and POT1 form a complex with telomeric DNA, which increases the activity and processivity of human telomerase. It was suggested that TPP1-POT1 switches from inhibiting telomerase access to the telomere as a component of Shelterin to working as a factor of telomerase processivity during the telomere elongation [201]. 

POT1 binds sing-strand telomeric DNA with a high specificity using two OB-motifs (oligonucleotide/oligosaccharide-binding folds). Without the 1st motif, the superexpression of POT1 leads to the quick elongation of telomeres [200]. Decreasing the hPOT1 expression can also lead to telomere elongation [202]. Another group of researchers has found that expressing the full-length protein leads to telomere elongation [203]. The binding of the recombinant POT1 with the telomere oligonucleotide inhibits the binding of telomerase. On the other hand, POT1 in vitro can destroy qudriplex structures, which are formed because of Hughstein pairing of nucleotides in telomeric DNA. This can explain how POT1 brings positive participation into the telomerase-dependent elongation of telomeres, because G-quadruduplexes seem like a very bad substrate for telomerase. The destruction of G-quadriduplexes could be also done by helicases WRN or BLM from the family RecQ, which react with POT1 [200]. In humans, mutations in genes that code helicases WRN or BLM lead to the development of the Blum and Werner syndromes, which are characterised by genomic instability [204].

Longer telomeres contain more Shelterin complexes, which could be a detector of the telomere length. Binding the protein POT1 with Shelterin can affect the binding of POT1 to the single-strand region of telomere DNA. Also, Shelterin can inhibit telomerase and facilitate T-loop formation, in which one of the 3-ends is not accessible [200].

The amount of POT1 mRNA in the case of stomach cancer often decreases in the early stages and increases at more advanced stages. The level of POT1 expression decreases according to the telomere shortening. Apart from this, the inhibition of POT1 in the stomach cancer cell lines by the anti-sense oligonucleotides, similarly to the inhibition of telomerase activity, also leads to telomeres shortening [205]. 

The binding of telomerase to the telomeres can be regulated by the formation/degradation of G-quadruduplexes. Single-stand DNA-binding protein RPA can untwist the telomeres G-quadruduplexes in the model systems [206]. On the other hand, RPA is able to inhibit telomerase activity in the model systems by binding with the telomere-imitating oligonucleotie [207]. The inhibition of telomerase in vitro occurs both after removing RPA from the mix and with its huge excess [208]. 

Unfortunately, the methods for analyzing telomerase activity use model systems with an artificial substrate without estimating access to the telomeres. 

### Regulation during the response to the ionising irradiation 

When investigating the processes that happen in oncogenesis, it is impossible to ignore the consequences of the effect of radiation from different sources on the cells. On the one hand, it can provoke oncogenesis (UV irradiation provokes skin cancer, penetrating ionising radiation can cause different types of cancers, etc.); on the other hand, different types of radiations are used in cancer therapy (β-particles, neutrons, γ and X rays, etc). Because the activity of telomerase is associated with a lot of different types of cancers, the following question occurs: what happens with this activity upon radiation? Which leads to another interesting question: could irradiation provoke telomerase activity?

While investigating the effect of ionizing irradiation on the HeLa cell line, it was found that telomerase activity and the amount of hTERT mRNA increase during the first 24 hours, until the twofold increase in comparison with the non-irradiated samples; however, after that they return to their original level within 72 hours [209].

After the application of γ and neutron/γ irradiation to the hemopoetic cell line, the increase in telomerase activity and in the amount of hTERT mRNA occurs in a dose- and intensity-dependent manner. In the case of neutron/γ irradiation, a decrease in telomerase activity occurs first, but it is followed by an increase. The influence of irradiation with different energies is different in scale and kinetics, but it is similar in the mechanism of its action on cells. Changes in telomerase activity are not related to either the changes in the cell cycle or the induction of cell death; they are a result of the specific regulator responses to ionizing irradiation [210].

HeLa cells with shortened telomeres-after transfection by DN-hTERT (dominant-negative mutant hTERT)-become more sensitive to the effect of several chemotherapeutic agents and irradiation. Cells transfected by the wild type of hTERT with longer telomeres show higher resistance to the chemotherapeutic agents and irradiation [211].

UV irradiation provokes telomerase activity in different types of cells, including skin cells. Apart from the skin, eyes are also permanently under UV irradiation. It was shown that the level of telomerase activity and amount of hTERT mRNA and hTR increase only after receiving a certain amount of UV-irradiation energy by crystalline lens [212].

### CONCLUSIONS

Knowing about the system of telomerase regulation allows us to create methods and agents for suppressing telomerase activity in cancer cells more effectively. Unfortunately, many mechanisms of telomerase regulation are tissue-specific. Understanding the interrelationship between the telomerase regulation system and other oncogenes can help in developing complex cancer diagnostics, which allows one to identify diseases and define the tactics for fighting them at the least aggressive stage. 

Telomerase activity is a marker of actively dividing cells and one of the most universal markers of cancer.
